# Harnessing therapeutic viruses as a delivery vehicle for RNA-based therapy

**DOI:** 10.1371/journal.pone.0224072

**Published:** 2019-10-23

**Authors:** Leena Ylösmäki, Beatrice Polini, Sara Carpi, Beatriz Martins, Elena Smertina, Sara Feola, Manlio Fusciello, Karita Peltonen, Paola Nieri, Erkko Ylösmäki, Vincenzo Cerullo

**Affiliations:** 1 Laboratory of Immunovirotherapy, Drug Research Program, Faculty of Pharmacy, University of Helsinki, Helsinki, Finland; 2 Laboratory of Molecular Pharmacology, Department of Pharmacy, University of Pisa, Pisa, Italy; Sechenov First Medical University, RUSSIAN FEDERATION

## Abstract

Messenger RNA (mRNA) and microRNA (miRNA)-based therapeutics have become attractive alternatives to DNA-based therapeutics due to recent advances in manufacture, scalability and cost. Also, RNA-based therapeutics are considered safe since there are no risk of inducing genomic changes as well as the potential adverse effects would be only temporary due to the transient nature of RNA-based therapeutics. However, efficient *in vivo* delivery of RNA-based therapeutics remains a challenge. We have developed a delivery platform for RNA-based therapeutics by exploiting the physicochemical properties of enveloped viruses. By physically attaching cationic liposome/RNA complexes onto the viral envelope of vaccinia virus, we were able to deliver mRNA, self-replicating RNA as well as miRNA inside target cells. Also, we showed that this platform, called viRNA platform, can efficiently deliver functional miRNA mimics into B16.OVA tumour *in vivo*.

## Introduction

Since the approval of the first oncolytic virus for the treatment of metastatic melanoma (Imlygic, a modified herpes simplex virus 1), therapeutic oncolytic viruses have gained great amount of interest in the field of immunovirotherapy. Recent clinical studies with Imlygic are showing that immune checkpoint inhibitor antibodies in combination with Imlygic significantly increase durable response rates in melanoma patients increasing the number of responders from 20% (as monotherapy) to as high as 50% [1]. However, the anti-tumour immune response elicited by the current oncolytic viruses used in the clinics remains modest and enhancement of this response might further increase the therapeutic efficacy of these viruses [2].

RNA-based therapeutics have become an attractive alternative to DNA-based therapeutics due to various beneficial properties including easier transfection process, higher efficiency and better safety profile[3]. MicroRNAs, small non-coding RNA molecules able to efficiently silence their target genes, have emerged as therapeutic targets for cancer [4]. Some miRNAs, able to target immune checkpoints, have been identified and they could be new potential immunotherapeutic drugs themselves [5]. Furthermore, miRNAs can target not only cancer and immune cells but also the tumour-promoting stromal cells, endothelial cells and tumour-associated fibroblasts, influencing many mechanisms involved in cancer progression [4]. MiRNA-based therapy can be used in two different ways: the re-introduction of down-regulated miRNAs with exogenous miRNA mimics, or the use of antagomirs for silencing endogenous miRNAs. MiRNA mimics, as small natural antisense oligonucleotides, have reduced immune response and lower toxicity compared to plasmid DNA-gene therapy and protein-based drug molecules [4]. Nevertheless, the *in vivo* delivery has some drawbacks, such as rapid blood clearance, enzymatic degradation and intracellular trapping into endosomes [4]. To improve the delivery of RNA-based therapeutics as well as to enhance the properties of current therapeutic enveloped viruses, we have developed a platform that can deliver broad range of functionally different RNA molecules into target cells by complexing the RNA molecules first with cationic liposomes followed by attaching this complex onto the viral envelope of vaccinia virus.

## Materials and methods

### Cell culture

Human lung carcinoma cell line A549 (ATCC, CCL-185) was cultured in DMEM with 10% foetal calf serum (FBS), 1% L-glutamine and 1% penicillin/streptomycin at 37°C/ 5% CO_2_. The cell line B16.OVA [6], a mouse melanoma cell line expressing chicken ovalbumin (OVA), was kindly provided by Prof. Richard Vile (Mayo Clinic, Rochester, MN, USA). B16.OVA cells were cultured in RPMI with 10% foetal calf serum (FBS), 1% L-glutamine, 1% penicillin/streptomycin and 1% geneticin at 37°C/ 5% CO_2_. Murine breast cancer cell line 4T1 (ATCC, CRL-2539) was cultured in RPMI-high glucose with 10% foetal calf serum (FBS), 1% L-glutamine and 1% penicillin/streptomycin at 37°C/ 5% CO_2_. Human melanoma cell line SK-MEL-2 (ATCC, HTB-68) was cultured in RPMI supplemented with 10% FBS, 1% penicillin/streptomycin and 1% L-glutamine. Baby hamster kidney cell line BHK-21 (ATCC, CCL-10) was cultured in MEM (Gibco) supplemented with 10% FBS, 1% penicillin/streptomycin, and 1% L-glutamine. All cells in culture were routinely tested for mycoplasma contamination with commercial detection kit (Lonza).

### Viruses

The Modified Vaccinia Ankara (MVA) virus was kindly provided by Prof. Gerd Sutter (Ludwig-Maximilians-University of Munich, Germany). MVA was produced in BHK-21 cells and purified by ultracentrifugation through 36% sucrose in 1 mM Tris (pH 9.0) cushion and eluted in 1 mM Tris (pH 9.0). The virus titre for the MVA was determined by Immunocytochemistry (ICC) staining. Briefly, BHK-21 cells were infected with 10-fold dilutions of virus in MEM with 2.5% FBS for 24 hours after which the cells were fixed with methanol/acetone (1:1) solution at -20°C for 5 min. Infected cells were stained with rabbit anti-vaccinia antibody (ab35219, Abcam) followed by incubation with HRP-anti-rabbit secondary antibody (ab6721, Abcam). The virus-infected cells were visualized with DAB substrate (Sigma Aldrich). Western reserve strain of vaccinia virus (VACV) coding for murine DNA-dependent activator of IFN-regulatory factors (mDAI) [7] was produced in A549 cells and purified through 36% sucrose cushion ultracentrifugation and eluted in 1mM Tris (pH 9.0). The virus titer for VACV was determined by plaque assay [8].

### RNA and RNA transcription

The Semliki Forest virus (SFV) self-amplifying replicon expressing ZsGreen was a kind gift from Dr. Tero Ahola (University of Helsinki, Finland). *In vitro* RNA transcription from SFV replicon was performed by using the SP6 RiboMAX Express Large-Scale RNA Production System (Promega). The template plasmids for enhanced green fluorescence (EGFP) and chicken ovalbumin mRNAs were ordered from GeneArt (Thermo Scientific). In vitro mRNA transcription was performed by using the T7 RiboMAX Express Large-Scale RNA Production System (Promega) according to the supplier's instructions. After the RNA synthesis, mRNAs were purified using the GeneJet RNA purification kit (Thermo Scientific).

### MiRNA mimics and transfection

miScript miRNA mimics of miR-17-5p, miR-34a-5p, miR-138-5p, miR‐193a‐3p and miR-200c-3p were purchased from Qiagen. The AllStar Negative Control siRNA i.e. scramble (Qiagen) has been used as a negative control in each experiment. Cell lines were plated to 60% confluence and transfected next day with 100 nM miRNA mimic or the negative control by using Lipofectamine 2000 (Invitrogen, Thermo Fisher Scientific, MA, USA), according to the manufacturer's instructions. Briefly, both Lipofectamine 2000 and miRNA mimics were diluted in Opti-MEM Medium (Euroclone, Milan, Italy) and complexed in 1:1 ratio (volume to volume). After 5 min of incubation, the complex was added to cells.

### viRNA complex formation

First, cationic liposome/RNA complexes were formed using Lipofectamine 2000 reagent as follows: 4 μl of Lipofectamine 2000 was diluted in 50 μl of Opti-MEM Reduced Serum Media (Gibco). After 5 min incubation at RT Lipofectamine was combined with 2 μg of RNA or 0.15 nmol or 0.5 nmol of miRNA mimic for *in vitro* and *in vivo* experiments, respectively, diluted in 50 μl of Opti-MEM and the mixture was then incubated 20 min at RT to allow cationic liposome/RNA complex to form. Cationic liposome/RNA complex was mixed with 2×10^6^ infectious units (IU) or 1×10^6^ plaque forming units (pfu) of virus (MVA or WR VACV, respectively) and the mix was incubated for 2 h on ice to allow the RNA to attach onto the viral envelope (viRNA complexes). After attachment, the unbound cationic liposome/RNA complexes were removed by centrifugation of the viRNA complexes through 36% sucrose cushion in 1mM Tris (pH 9.0) for 1 h at 20.000g, at 4°C and the pellet was resuspended in PBS. In the case of RNaseA-treated viRNA- enhanced green fluorescent protein (EGFP), 200 μg of RNaseA was added to the viRNA complex solution and incubated for 10 minutes at RT followed by removal of the unbound RNA-lipofectamine complexes by centrifugation of the complex through 36% sucrose cushion in 1mM Tris (pH 9.0) for 1 h at 20.000g, at 4°C followed by resuspension in PBS.

### Zeta potential analysis of the viRNA complexes

VirNA-mEGFP WR and viRNA-mEGFP MVA complexes were prepared as described in previous section. In addition, both viral strains (WR and MVA) alone or complexed with only lipofectamine were used as controls. For measuring the zeta potential of different complexes, each sample was diluted to a final volume of 700 μL with sterile Milli-Q water adjusted to pH 7.4 and transferred to a DTS1070 disposable capillary cell (Malvern, Worcestershire, UK) for zeta potential measurements. All measurements were performed at 25°C with a Zetasizer Nano ZS (Malvern).

### Nanoparticle tracking analysis of the viRNA complexes

VirNA-mEGFP WR and viRNA-mEGFP MVA complexes were prepared as described in viRNA complex formation- section. In addition, both viral strains (WR and MVA) alone or complexed with only lipofectamine were used as controls. For size determination, all samples were diluted 1/10 with 0.9% sodium chloride before nanoparticle tracking analysis. All size measurements were performed with Nanosight NS300 nanoparticle tracking analyser (Malvern) at 25°C. For stability determination, viRNA-mEGFP MVA complexes were prepared as described earlier and nanoparticle tracking analysis and infection of A549 cells was performed directly after sucrose cushion purification of the complexes or prior nanoparticle tracking analysis and infection of A549 cells, complexes were left on ice for 30, 60 or 120 minutes.

### Flow cytometric analysis of PD-L1 expression

The cells transfected with miRNA-mimic or infected with miRNA-mimic complexed with MVA (viRNA-mimic MVA) were stained with anti-PD-L1 (PE-CD274, eBioscience) antibody after 48 h of transfection or infection. Flow cytometric analyses of the expression level of PD-L1 were performed by using BD Accuri 6C plus flow cytometer (BD Biosciences) and data were analysed with FlowJo software v10 (Tree Star, Ashland, OR, USA).

### Generation of bone marrow-derived dendritic cells (BMDCs)

2×10^6^ bone marrow cells isolated from C57BL/6JOlaHsd mouse were seeded in 10 ml complete medium RPMI containing 10 ng/ml recombinant murine granulocyte–macrophage colony-stimulating factor (PeproTech), 10% foetal calf serum (Sigma), 2 mM L-glutamine (Gibco) and 1% penicillin/streptomycin. Cells were cultured at 37°C/ 5% CO^2^. On the day 3, 10 ml of complete medium was added and on the days 6 and 8, 9 ml of media was gently aspirated and replaced with 10 ml of fresh complete medium. Following 10 days of culture, BMDCs were harvested and used for cross-presentation experiments described below.

### Cross-presentation experiments

For cross-presentation experiments, 1×10^6^ BMDCs were infected with multiplicity of infection (MOI) 1 of viRNA-mOVA WR for 24 hours. The cells were collected and stained with APC-conjugated anti-mouse H-2Kb bound to SIINFEKL (141606, BioLegend) or APC-conjugated Mouse IgG1, κ Isotype Ctrl (400119, BioLegend) together with FITC-conjugated CD11c (557400, BD), PerCP/Cy5.5-conjugated CD86 (105028, BioLegend), and PE-conjugated CD103 (121406, BioLegend). The samples were analysed with BD Accuri C6 plus flow cytometer (BD Biosciences) and the FlowJo software v10 (FlowJo, LLC) was used for the data analysis.

### Animal experiments

All animal experiments were reviewed and approved by the Experimental Animal Committee of the University of Helsinki and the Provincial Government of Southern Finland (license number ESAVI/9817/04.10.07/2016). Animals (n = 25) were kept in individually ventilated cages (5 mice/cage) under standard conditions (12h light:dark, temperature- and humidity-controlled conditions) and received ad libitum access to water and food. Animals where monitored daily for symptoms related to distress and pain including hunched posture, overall activity/ability to move and roughness of the hair coat. Tumour dimensions were measured by using calliper (vertical and lateral dimensions) every second day, starting at day seven. Tumour measurements and intratumoural injections were performed under isoflurane anaesthesia. Animals were sacrificed before either vertical or lateral dimension reached the maximum allowed (17mm) using terminal isoflurane anaesthesia followed by cervical dislocation. Female C57BL/6JOlaHsd mice (Envigo, UK) were injected in the right flank with 350,000 B16.OVA melanoma cells. When the average tumour size reached approximately 60 mm^3^ (11 days after injection), each mouse was treated with 10^6^ IU of viRNA mimic MVA. In detail, mice were treated with viR-138-5p MVA (0.5 nmol of miR-138-5p mimic complexed with MVA), viR-193a-3p MVA (0.5 nmol of miR-193a-5p mimic complexed with MVA) or viR-138-5p/193a-3p MVA (viRNA with a combination of miR-138-5p and miR-193a-3p with 0.25 nmol of each miRNA mimic complexed with MVA). Mice injected with 10^6^ IU of MVA complexed with siRNA-scramble (viR-scramble) were used as control and mice injected with PBS only were used as a mock-treated group. Groups of five mice were treated intratumourally on days 11 and 14 post tumour implantation. All mice were sacrificed on day 17 and tumours were collected and analysed for PD-L1 expression by flow cytometry with BD Accuri C6 plus flow cytometer (BD Biosciences).

### Statistical analysis

Statistics were performed using GraphPad Prism 7.0 (GraphPad Software, San Diego, CA, USA). Unpaired Student’s t-test, one-way or two-way ANOVA with Tukey’s multiple comparison test or Dunnett’s multiple comparison test were used.

## Results

### Enveloped viruses can be transfected with RNA molecules of various size and function

**Fig 1:**
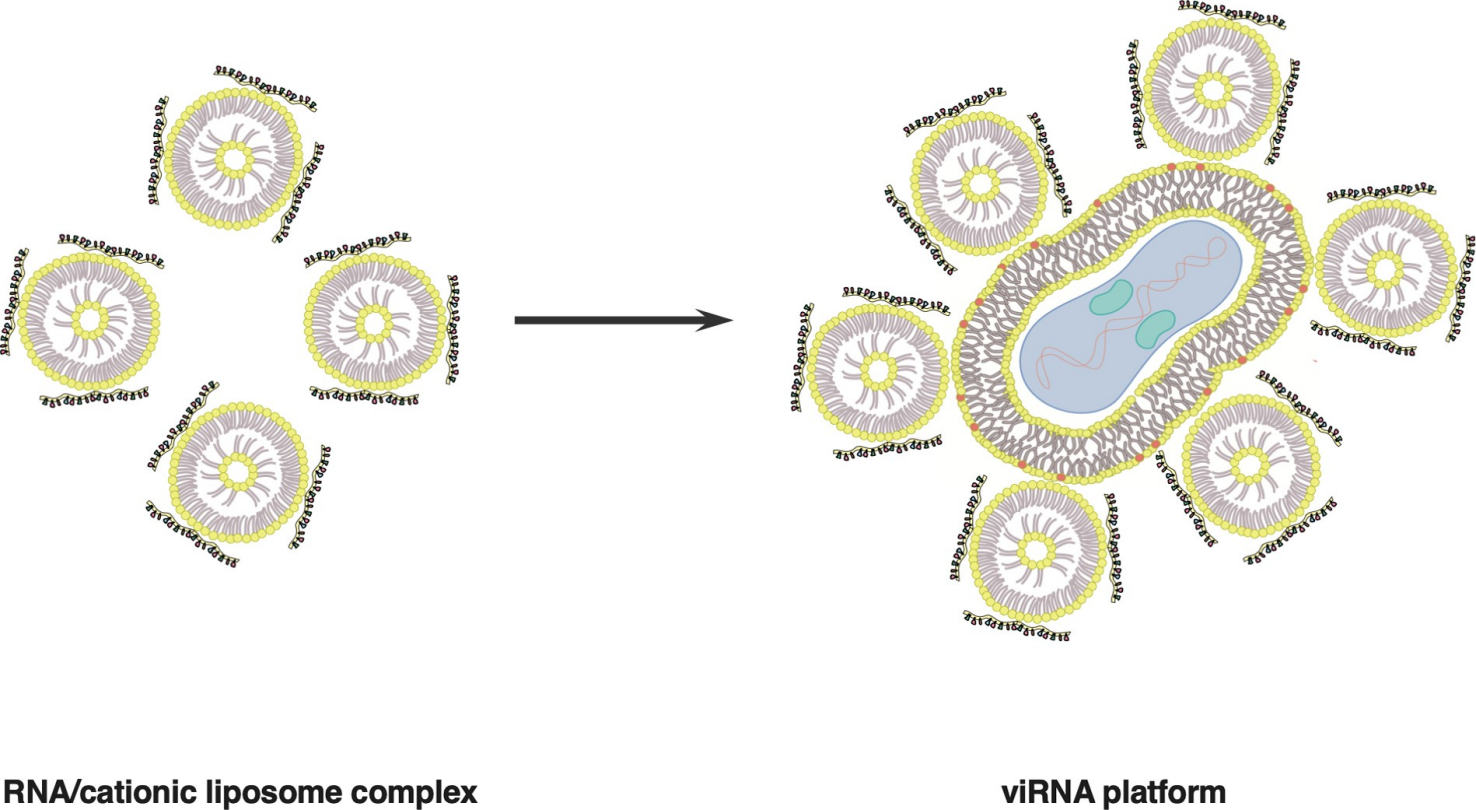
A schematic presentation of viRNA RNA-delivery platform. RNA molecules of various size and function can be attached onto enveloped viruses by using cationic liposomes. RNA molecules are first complexed with cationic liposomes and after the complex formation, the RNA/cationic liposome complex is mixed with enveloped virus to allow the attachment of the complex with the viral envelope. After attachment, viRNA complexes are purified for unbound RNA/cationic liposome complexes by centrifugation through 36% sucrose cushion for 1h at 20 000g.

**Fig 2:**
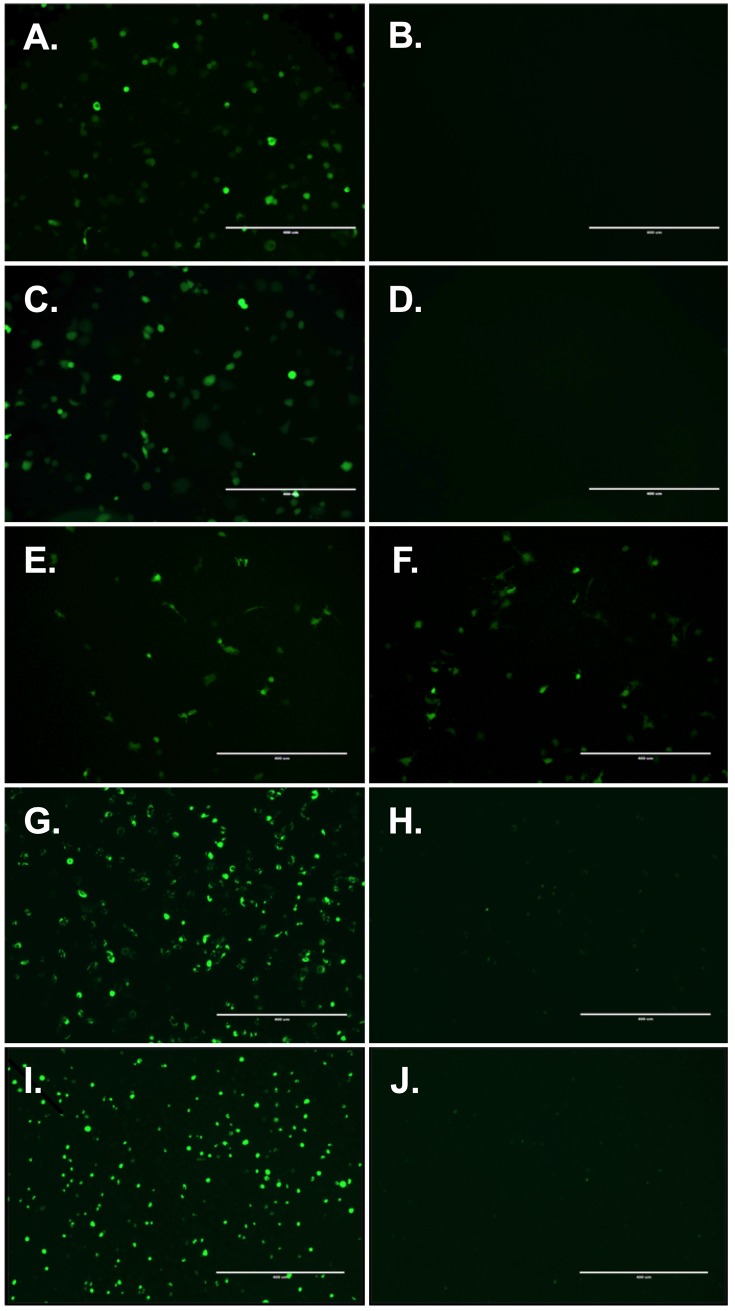
In vitro characterization of the viRNA platform. A) Sucrose purified viRNA-mEGFP WR was used to infect A549 cells and imaged for EGFP expression after 24h post-infection. B) Vaccinia WR strain was incubated with mRNA of EGFP without cationic liposomes and sucrose purified prior infection to assess the unspecific interaction between vaccinia and mRNA of EGFP. C) Sucrose purified viRNA-mEGFP MVA was used to infect A549 cells and imaged for EGFP expression after 24h post-infection. D) Cationic liposomes were complexed with mRNA of EGFP without interacting with vaccinia and sucrose purified prior transfection to assess the efficacy of the sucrose purification on the viRNA complexes. E) viRNA-mEGFP WR was used to infect A549 cells without RNaseA-treatment and in F) viRNA-mEGFP WR was treated with RNaseA prior infection of A549 cells. G) Sucrose purified viRNA-SA-ZsGreen WR was used to infect A549 cells and imaged for EGFP expression after 24h post-infection. H) Cationic liposomes were complexed with SA-ZsGreen without interacting with vaccinia and sucrose purified prior transfection and imaged for EGFP expression after 24h post-transfection. I) viRNA-SA-ZsGreen WR was used to infect A549 cells and imaged for EGFP expression after 4 days post-infection. J) Cationic liposomes were complexed with SA-ZsGreen without interacting with vaccinia and sucrose purified prior transfection and imaged for EGFP expression after 4 days post-transfection.

As the outer membrane of all enveloped viruses consist of lipid bilayer derived from the host cell, we hypothesized that cationic liposomes carrying various different RNA molecules could be transfected onto an enveloped virus (see Fig 1 for schematic presentation of the viRNA-platform). First, we tested messenger RNA (mRNA) of EGFP complexed with cationic liposomes and transfected either vaccinia virus strain western reserve (WR) or modified vaccinia Ankara (MVA). As seen in Fig 2A for viRNA-mEGFP WR and Fig 2C for viRNA-mEGFP MVA, both strains can be efficiently attached by cationic liposomes carrying mRNA of EGFP. As a control, we tested whether there are unspecific interactions between the viral envelope and mRNA and allowed the complexation of these two components to occur without cationic liposomes (Fig 2B). Also, we complexed cationic liposomes with mRNA of EGFP but did not allow the complex to interact with viral envelope (Fig 2D). As we were able to see only very few, if any, EGFP-positive cells after infection/transfection with these control reactions, we confirmed the specific interaction between viral envelope and liposomes carrying mRNA of EGFP. Next, we assessed whether the mRNA molecules in the viRNA complex are shielded and protected against external RNases. To test that, we made viRNA-mEGFP WR complex and incubated half of the reaction briefly with RNaseA prior complex purification and infection of the cell monolayer. The number of EGFP-positive cells infected with non-treated (Fig 2E) or RNaseA-treated (Fig 2F) viRNA-mEGFP WR were very similar indicating that RNA may be shielded against external RNases when in viRNA complex. We also wanted to see whether we could introduce larger RNA molecules into the viRNA-platform and whether we could have more prolonged and more robust expression of green fluorescent protein by using self-amplifying RNA molecule of the size of 8.2 kilobases. A viRNA complex was prepared using self-amplifying RNA molecule expressing ZsGreen (viRNA-SA-ZsGreen WR) and viRNA-SA-ZsGreen WR-infected cells were monitored for 4 days. As seen in Fig 2G and Fig 2I, viRNA-platform can accommodate long RNA molecules and the self-amplifying RNA was very well expressed after four days post-infection.

### Biophysical characterization of viRNA complexes

**Fig 3:**
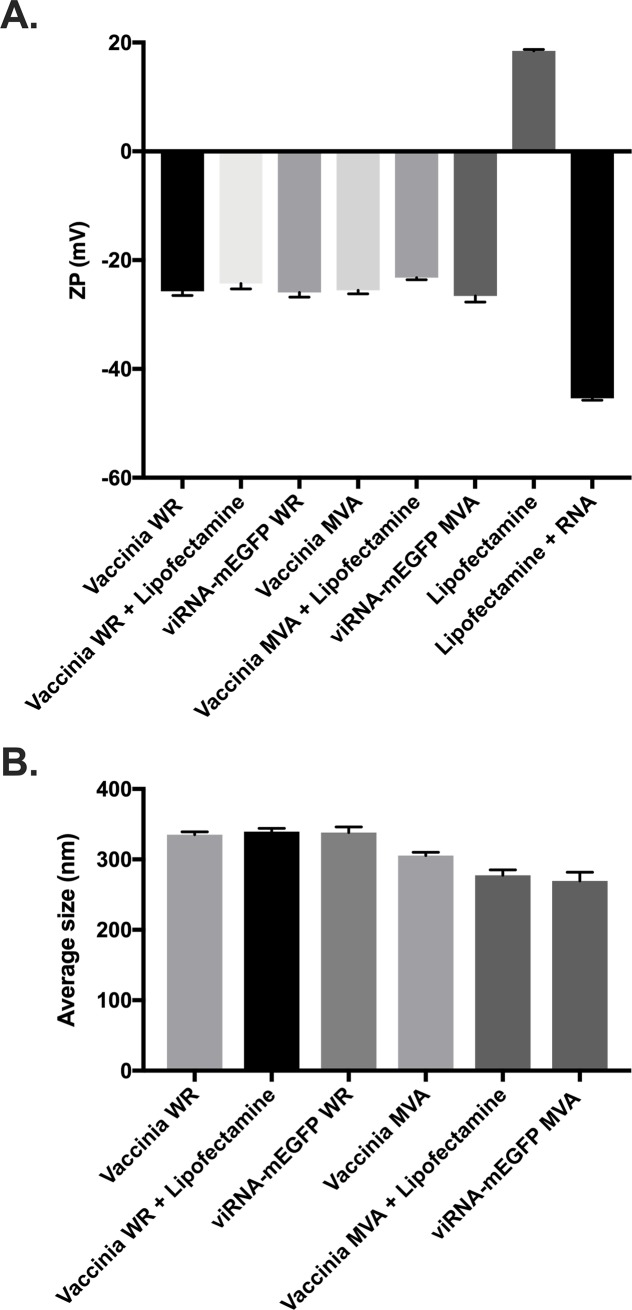
Determination of Zeta potential and average size of viRNA complexes. A) Zeta potential measurements of vaccinia WR and MVA strains and viruses complexed with either only lipofectamine or lipofectamine together with mRNA of EGFP as in viRNA-mEGFP WR and viRNA-mEGFP MVA. Lipofectamine alone and lipofectamine complexed with mRNA of EGFP were used as controls. B) Nano tracking analysis derived average size of vaccinia WR and MVA strains and viruses complexed with either only lipofectamine or lipofectamine together with mRNA of EGFP as in viRNA-mEGFP WR and viRNA-mEGFP MVA. Each bar is the mean ± SEM of technical pentaplicates.

**Fig 4:**
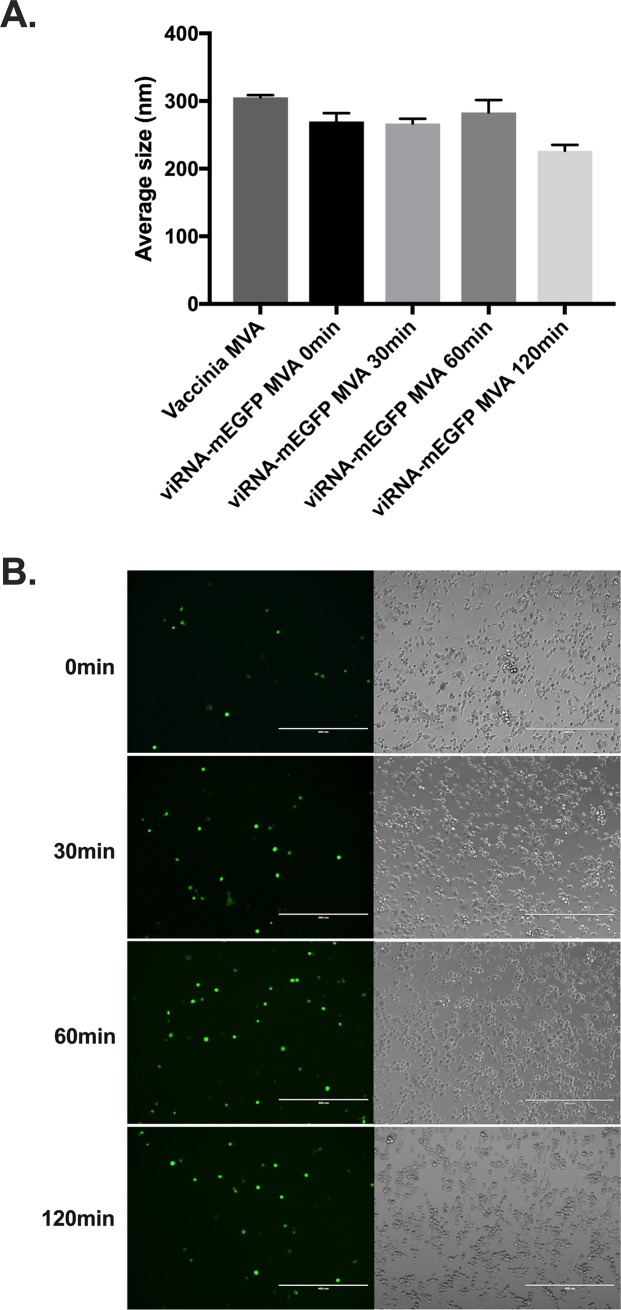
viRNA complex stability determination. A) Sucrose purified viRNA-mEGFP MVA complexes were incubated on ice for extended length of time and in A) the average size of the complex was determined at indicated time points and in B) the complexes were used to infect A549 cells for the assessment of complex stability and functionality. Each bar in A is the mean ± SEM of technical pentaplicates.

For understanding the biophysical characteristics of the viRNA complex, we determined Zeta potential, size and stability of these complexes. As seen in Fig 3A, both viRNA-mEGFP WR and viRNA-mEGFP MVA have similar Zeta potential as vaccinia WR and MVA, respectively. Nanoparticle tracking analysis was used to determine the average size of viRNA-mEGFP WR and viRNA-mEGFP MVA complexes (Fig 3B). The average size of both viRNA-mEGFP WR and viRNA-mEGFP MVA were similar to the average size of vaccinia WR and MVA, respectively. For the assessment of short-term stability and aggregation of the viRNA complexes, we incubated viRNA-mEGFP MVA complexes on ice for up to 120 minutes prior to size determination and infection of A549 cells. ViRNA-mEGFP MVA complexes did not show marked signs of aggregation even after elongated amount of time incubated on ice (Fig 4A and see also S1 Fig for complete finite track length adjustment (FTLA) concentration/size graphs for the nanoparticle tracking analysis). We also infected A549 cells with these complexes to see whether they remain functional after incubation on ice for up to 120 minutes. As shown in Fig 4B, viRNA-mEGFP MVA complexes remain functional at all time points tested as assessed by the number of EGFP-positive viRNA-mEGFP MVA-infected cells.

### Antigen presenting cells can efficiently present antigens delivered by viRNA-platform

**Fig 5:**
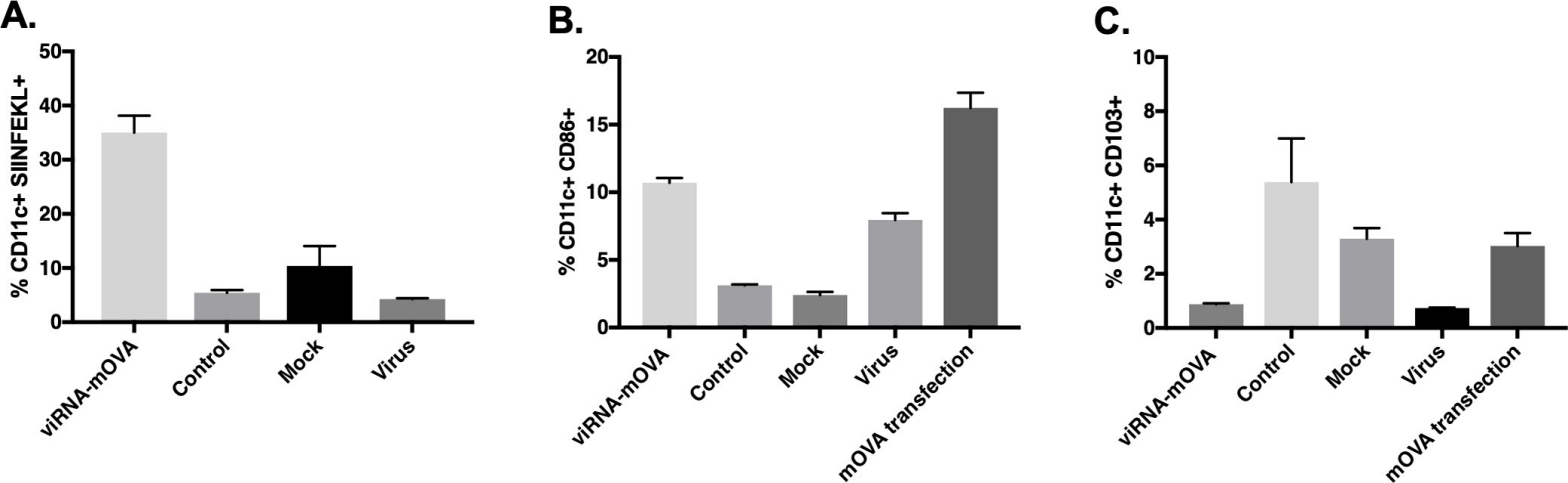
Antigen presentation and activation of bone marrow-derived dendritic cells after infection with viRNA-mOVA. Mouse bone marrow-derived dendritic cells were infected with sucrose purified viRNA-mOVA WR, sucrose purified mOVA complexed with cationic liposomes as a control, virus alone or non-treated cells as a mock. A) Antigen presentation was determined by flow cytometry using APC-conjugated anti-H-2Kb bound to SIINFEKL. B) Dendritic cell activation and C) migration marker expression was determined by flow cytometry using antibodies against murine CD86 and murine CD103, respectively. Each bar is the mean ± SEM of technical triplicates.

We went on to test whether viRNA-platform could be used as a platform for vaccine development. To this end, we used mRNA coding for chicken ovalbumin (OVA) and complexed it with cationic liposomes and transfected vaccinia WR strain with the complex to obtain viRNA-mOVA WR. We infected mouse bone marrow-derived dendritic cells (BMDCs) with this viRNA-mOVA WR to assess whether viRNA-platform enables functional mRNA to be translated, processed and presented on major histocompatibility complex class I (MHC-I) molecules by the antigen presenting cells. We also assessed the activation and maturation of the BMDCs and compared these characteristics with virus infection alone as well as normal cationic liposome transfection of mRNA OVA. As shown in Fig 5A, infection of BMDCs with viRNA-mOVA WR led to an efficient mRNA processing and MHC-I presentation of epitopes as assessed by the presentation of the immunodominant epitope of ovalbumin (SIINFEKL). Interestingly, viRNA has similar characteristics in dendritic cell activation and maturation as the backbone virus rather than the cationic liposomes used in transfection. Both viRNA-OVA and virus groups showed similar, modest upregulation of CD86, a marker for dendritic cell maturation, whereas mRNA OVA transfection of BMDCs with cationic liposomes showed markedly increased upregulation of CD86 (Fig 5B). Similarly, both viRNA-OVA WR and virus groups showed similar downregulation of CD103, a marker for dendritic cell migration, whereas mOVA transfection had no effect on the expression of CD103 (Fig 5C).

### miRNA mimics delivered by the viRNA-platform show significant down-regulation of their target proteins

**Fig 6:**
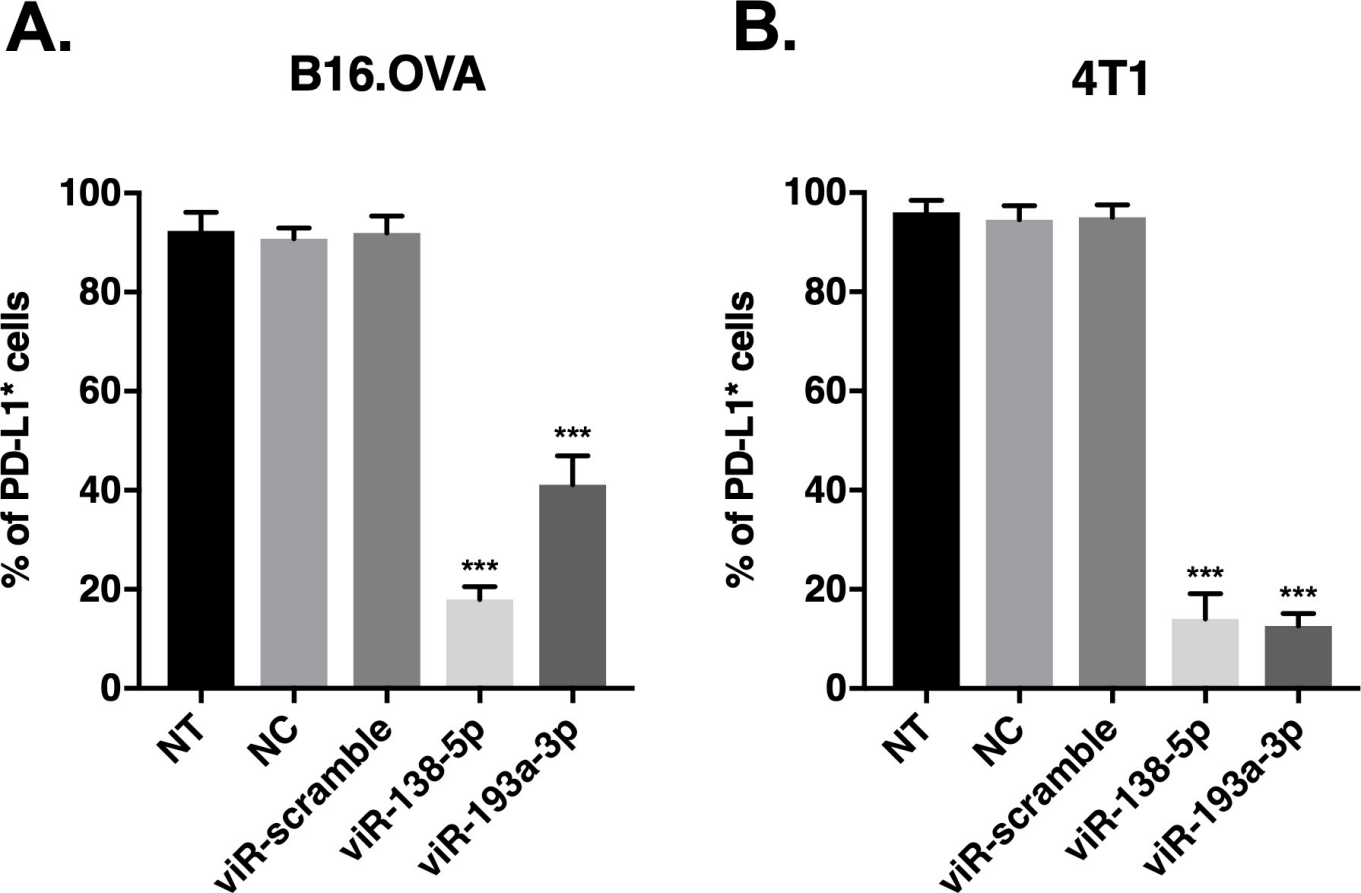
PD-L1 targeting MiRNA mimics delivered by viRNA platform induce efficient down-regulation of PD-L1 expression in murine melanoma and breast cancer cell lines. A) B16.OVA cells and B) 4T1 cells were infected with viR-138-5p, viR-193a-3p or viR-scramble and 48 hours post-infection, cells were harvested and PD-L1 protein levels were analysed by flow cytometry using antibody against murine PD-L1. Data shown as a mean ± SEM of three independent experiments performed in triplicate. One-way ANOVA followed by Dunnett’s multiple comparison test. *** p-value < 0.001 compared to viR-scramble; (NT, non-treated cells; NC, negative control: sucrose purified miR-138-5p/lipofectamine complex).

Next, we tested the efficiency of viRNA-delivered miRNAs to downregulate their target molecules in various cell lines. First, we screened multiple miRNAs that have been reported to down-regulate programmed cell death ligand-1 (PD-L1) and chose miR-138-5p and miR-193a-3p as they exhibited the most potent down-regulation of PD-L1 in both B16.OVA and 4T1 murine cell lines (see S2 Fig). We selected miR-138-5p and miR-193a-3p that were shown to efficiently down-regulate PD-L1 protein expression and complexed them with cationic liposomes and transfected vaccinia MVA strain with the complex to obtain viR-138-5p MVA and viR-193a-3p MVA, respectively. As shown in Fig 6, infection of either viR-138-5p MVA or viR-193a-3p MVA induced a significant decrease in the number of cells expressing PD-L1 in both cell lines tested as compared to control viRNA containing scramble siRNA (viR-scramble) or sucrose purified liposomes complexed with miR-138-5p (as a negative control, NC). Furthermore, the down-regulation of PD-L1 by viRNA system was comparable with PD-L1 down-regulation by transfection with lipofectamine 2000 in both cell lines. The number of PD-L1-expressing B16.OVA cells after transfection with miR-138-5p and miR-193a-3p was decreased to 32.2% and 39.6 ± 9.0% as compared to control cells, respectively. Similarly, in B16.OVA cells infected by viR-138-5p MVA and viR-193a-3p MVA the number of PD-L1-expressing B16.OVA cells was decreased to 21.7% and 44.7% as compared to control cells, respectively. The same trend was observed in 4T1 cells, where transfection with miR-138-5p or miR-193a-3p decreased the number of cells expressing PD-L1 to 7.6% and 4.3% as compared to control cells, respectively. A comparable down-regulation of PD-L1 was observed in these cells when infected with viR-138-5p MVA or viR-193a-3p MVA as the number of PD-L1 positive cells decreased to 14.8% and 13.2% as compared to control cells, respectively.

**Fig 7:**
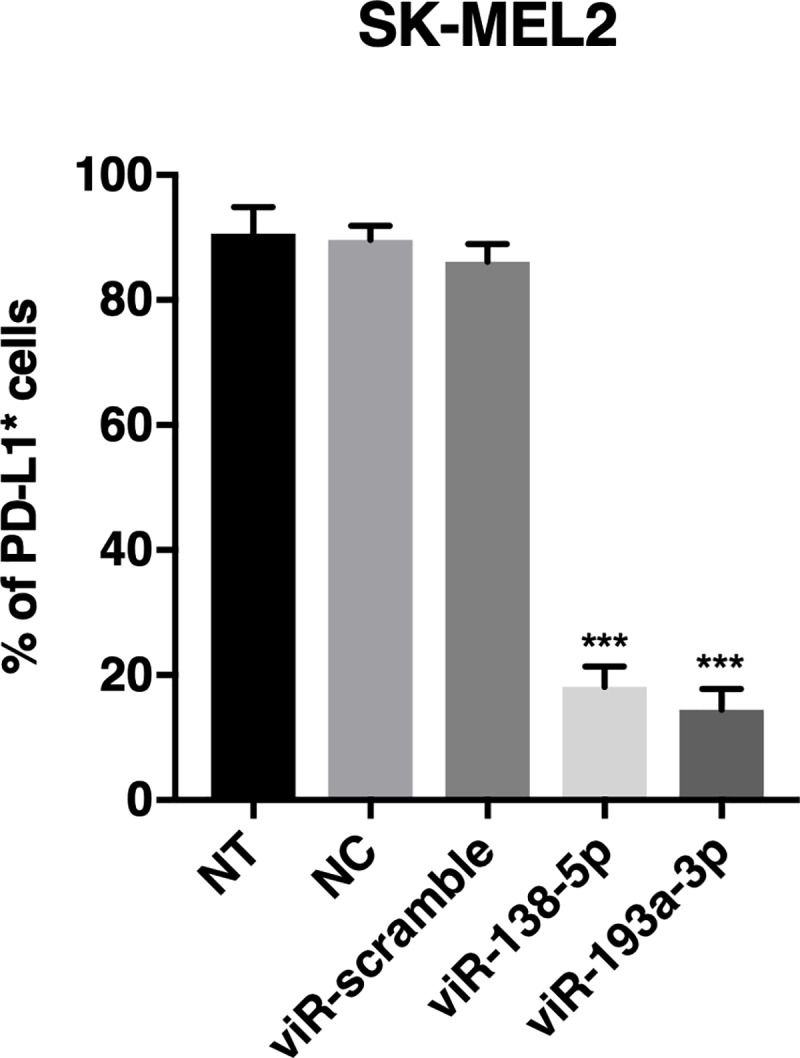
PD-L1 targeting MiRNA mimics delivered by viRNA platform induce efficient down-regulation of PD-L1 expression in human melanoma cell line SK-MEL2. SK-MEL2 cells were infected with viR-138-5p, viR-193a-3p or viR-scramble and 48 hours post-infection, cells were harvested and PD-L1 protein levels were analysed by flow cytometry using antibody against human PD-L1. Data shown as a mean ± SEM of three independent experiments performed in triplicate. One-way ANOVA followed by Dunnett’s multiple comparison test. *** p-value < 0.001 compared to viR-scramble; (NT, non-treated cells; NC, negative control: sucrose purified miR-138-5p/lipofectamine complex).

In order to evaluate the efficiency of viRNA-platform to down-regulate PD-L1 protein expression in a human *in vitro* model, viR-138-5p MVA and viR-193a-3p MVA were also tested in a human melanoma cell line SK-Mel-2. To compare the efficiency to down-regulate human PD-L1 of viR-138-5p MVA and viR-193a-3p MVA to miR-138-5p and miR-193a-3p transfection by lipofectamine 2000, SK-Mel-2 cells were transfected with miR-138-5p or miR-193a-3p using lipofectamine 2000 or infected with viR-138-5p MVA or viR-193a-3p MVA. After 48h, cells were analysed by flow cytometry to evaluate the number of cells expressing PD-L1. Transfection with lipofectamine 2000 confirmed the ability of the two selected miRNAs to decrease the number of SK-Mel-2 cells expressing PD-L1 (see S2 Fig). After transfection with miR-138-5p and miR-193a-3p, the number of cells expressing PD-L1 were 16.3% and 18.6% as compared to control cells, respectively. Similarly, cells infected with viR-138-5p MVA and viR-193a-3p MVA, the number of cells expressing PD-L1 were 21.1% and 16.8% as compared to control cells, respectively (Fig 7). These data suggest that viRNA-platform is able to efficiently deliver functional miRNAs in to the target cells *in vitro*.

### viRNA platform can efficiently down-regulate cancer cell PD-L1 expression in syngeneic mouse model of B16.OVA melanoma

**Fig 8:**
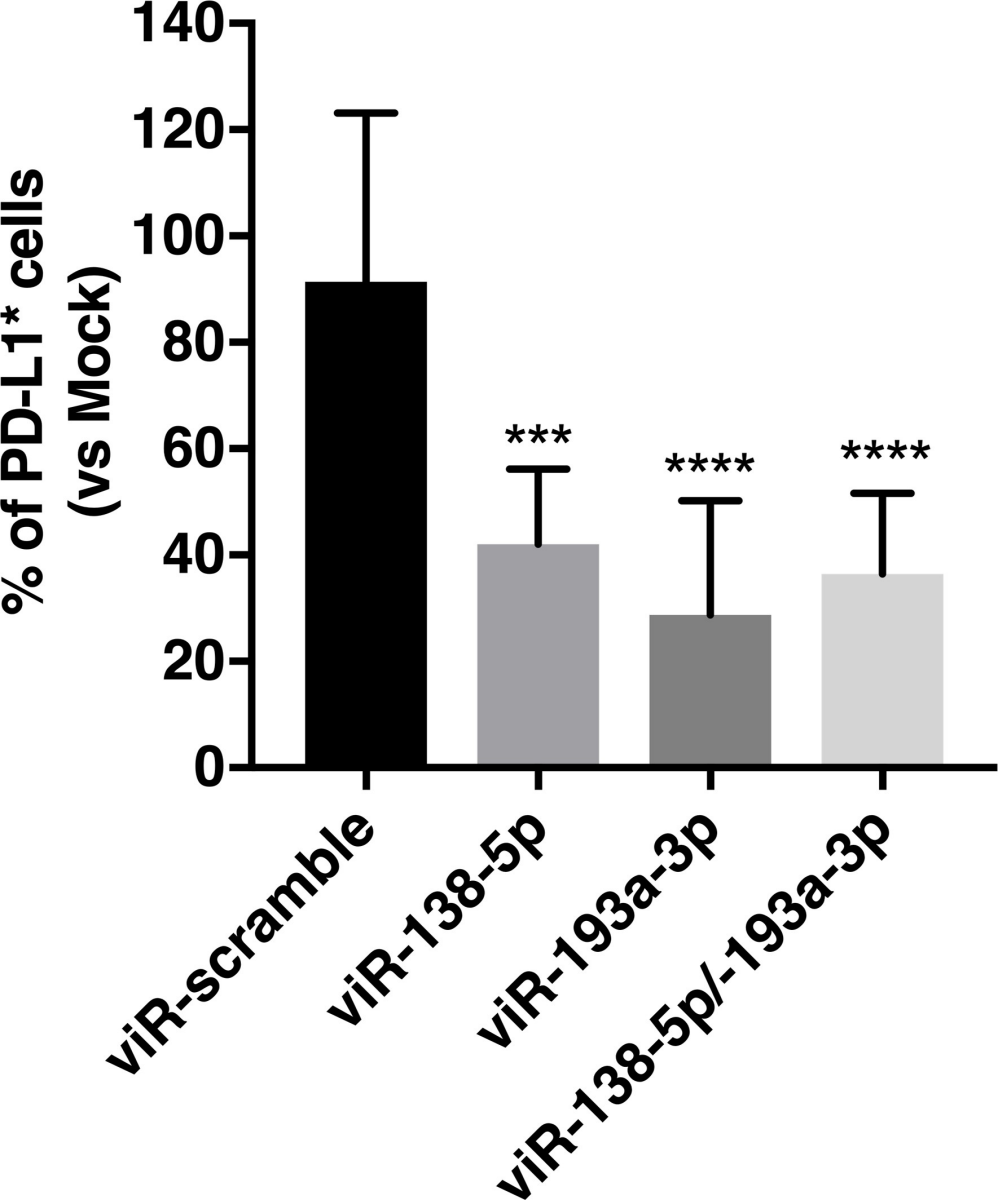
MiRNA mimics delivered intratumourally with viRNA platform enables efficient down-regulation of cancer cell-expressed PD-L1. B16.OVA tumour-bearing mice were treated intratumourally with either viR-138-5p, viR-193a-3p, viR-138-5p/193a-3p or viR-scramble as a control on days 11 and 14 post-tumour implantation and mice were sacrificed and tumours collected on day 16. PD-L1 protein levels were analysed by flow cytometry using antibody against human PD-L1. Data shown as a mean ± SEM. One-way ANOVA followed by Dunnett’s multiple comparisons test. *** p-value < 0.001 and **** p-value < 0.0001 compared to viR-scramble.

Finally, we tested the efficacy of the viRNA platform to deliver miRNAs in *in vivo* settings using syngeneic mouse model of B16.OVA melanoma. Mice bearing B16.OVA tumours were treated intratumourally on days 11 and 14 after tumour implantation with viR-138-5p MVA, viR-193a-3p MVA, viR-138-5p/193a-3p MVA (viRNA with a combination of miR-138-5p and miR-193a-3p) or viR-scramble MVA as a control. As shown in Fig 8, both viR-138-5p MVA and viR-193a-3p MVA were able to significantly down-regulate PD-L1 expression in the tumour as compared to viR-scramble MVA and mock-treated animals.

Also, the viRNA complexed with both miR-138-5p and miR-193a-3p showed similar efficacy as viR-138-5p MVA and viR-193a-3p MVA, suggesting the possible use of the viRNA platform to deliver simultaneously more than one miRNA.

## Discussion

A great limitation for therapeutic use of RNAs, including miRNAs, is the difficulty of the *in vivo* delivery. Due to the physicochemical properties of RNA molecule itself, it is incapable of crossing the cell membrane independently, thus a suitable delivery vector, either viral or non-viral, must be used to achieve adequate delivery in to the target cells [3]. In this study, we have developed a platform for enhancement of cancer immunovirotherapy by exploiting the physicochemical properties of enveloped viruses and showed that RNA molecules of various sizes and functionalities complexed with cationic liposomes can be physically attached onto the viral envelope for efficient intracellular delivery. We first characterized the platform with differently sized RNA molecules coding for green fluorescent protein and showed that both mRNA molecules and self-amplifying RNA molecules can be loaded into the viRNA platform and efficiently delivered into target cells. Next, we characterized the Zeta potential and size of the viRNA complexes and interestingly, both the Zeta potential and size of the viRNA complexes used in the characterization did not significantly differ from naked vaccinia viruses. One explanation for this could be that the liposome:RNA:virus ratio in the complex preparation was not optimal, thus the number of viRNA complexes in the preparation was not high enough to significantly alter the Zeta potential or average size as compared to the naked viruses.

We also examined whether viRNA platform could function as a cancer vaccine platform and showed that mRNA of chicken ovalbumin (used as a model antigen) could be delivered into antigen presenting cells by the viRNA platform, and consequently the infected antigen presenting cells were shown to efficiently present the immunodominant epitope of the ovalbumin. As miRNA-based therapy is a promising approach for future anti-cancer treatment, but still encounter several barriers such as poor bioavailability, limited tissue permeability and payload instability as well as inefficient *in vivo* delivery [4], we set out to test whether viRNA platform could be suitable for miRNA-based anti-cancer therapy. To test this, we chose miRNA mimics for miR-138-5p and miR-193a-3p, shown to down-regulate PD-L1 protein, to be used with the viRNA platform. Remarkably, both viR-138-5p MVA and viR-193a-3p MVA showed very potent down-regulation of PD-L1 in all cell lines tested. Also, intratumoural treatment of B16.OVA tumour-bearing mice with viR-138-5p MVA and viR-193a-3p MVA as well as viR-138-5p/193a-3p MVA showed significant down-regulation of PD-L1 expression. Indeed, the observed down-regulation of PD-L1 protein expression in viRNA-treated mice indicate that miRNA mimics were efficiently delivered into tumour cells and maintained their integrity and functionality.

Another feature of the viRNA platform to be highlighted is the ability to simultaneously deliver multiple different miRNAs. This ability could be very beneficial as it may allow using a combination of different miRNAs to down-regulate different signalling pathways simultaneously to obtain most prominent anti-cancer effect. Since the heterogeneity of cancer is linked to simultaneous alterations of several pathways, the delivery of miRNA combinations may be an effective way to enhance the treatment of cancer [9]. Moreover, the control of multiple pathways is an efficient strategy to counteract occurrence of resistance. In the current study, we used non-replicating vaccinia virus strain MVA in the *in vivo* experiment to more clearly assess the delivery and efficacy of the miRNA mimics on down-regulating the PD-L1 protein expression. Using viRNA platform with replication-competent viruses to deliver therapeutic miRNAs or mRNAs coding e.g. for cancer antigens into the tumour microenvironment might potentiate the oncolytic virotherapy and enhance the immunotherapeutic potential of the used enveloped viruses. It could also allow the development of more personalized oncolytic virus cancer vaccine approaches if one could introduce patient-specific mRNAs coding for neo-antigens in to the viRNA platform.

## Supporting information

S1 Fig Determination of the stability of viRNA complexes. Averaged finite track length adjustment (FTLA) concentration/size graphs for the nanoparticle tracking analysis. A) Vaccinia MVA in Optimem media. B) Sucrose purified vaccinia MVA. C) Lipofectamine 2000/mRNA of EGFP complex without virus. D) viRNA-mEGFP MVA complex prior sucrose purification. Sucrose purified viRNA-mEGFP MVA complex in E) no incubation on ice after purification or after F) 30 minutes G) 60 minutes and H) 120 minutes incubation on ice. Error bars in red indicate +/-1 standard error of the mean.
(TIFF)

S2 Fig **Effect of selected miRNA mimics on PD-L1 expression in 4T1 (A), B16.OVA (B) and SK-Mel2 (C).** B16.OVA cells and 4T1 cells were transfected with miR-17-5p, miR-34a-5p, miR-138-5p, miR-193a-3p or miR-200c-3p and 48 hours post-infection, cells were harvested and PD-L1 protein levels were analysed by flow cytometry using antibody against murine PD-L1. SK-MEL2 cells were transfected with miR-138-5p, miR-193a-3p or miR-scramble as a control and 48 hours post-infection, cells were harvested and PD-L1 protein levels were analysed by flow cytometry using antibody against human PD-L1. Data are presented as mean ± SEM of three independent experiments performed in triplicate. One-way ANOVA followed by Dunnett’s multiple comparisons test. ** p-value < 0.01 compared to control; *** p-value < 0.001 compared to control (NT, non-transfected cells).
(TIFF)

S1 Table miRNAs selected from literature for their ability to down-regulate PD-L1 in different types of human cancer. (TIFF)
