# Supplementary figures and images for: Harnessing therapeutic viruses as a delivery vehicle for RNA-based therapy
Source: PLoS One. 2019 Oct 23;14(10):e0224072. doi: 10.1371/journal.pone.0224072 (PMC6808555; doi:10.1371/journal.pone.0224072)

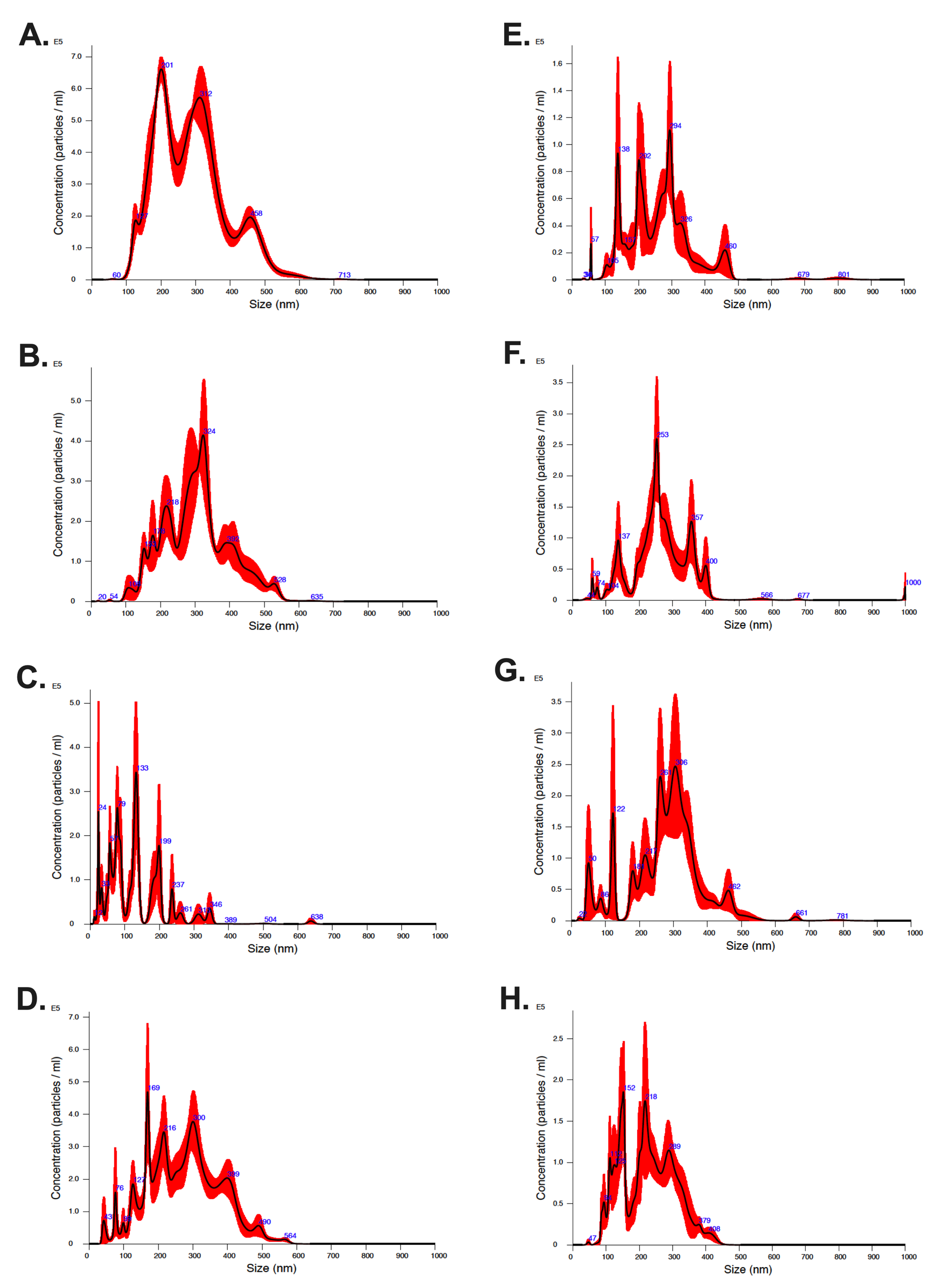

Supplement: S1 Fig — Averaged finite track length adjustment (FTLA) concentration/size graphs for the nanoparticle tracking analysis. A) Vaccinia MVA in Optimem media. B) Sucrose purified vaccinia MVA. C) Lipofectamine 2000/mRNA of EGFP complex without virus. D) viRNA-mEGFP MVA complex prior sucrose purification. Sucrose purified viRNA-mEGFP MVA complex in E) no incubation on ice after purification or after F) 30 minutes G) 60 minutes and H) 120 minutes incubation on ice. Error bars in red indicate +/-1 standard error of the mean. (TIFF) [file pone.0224072.s001.tiff]

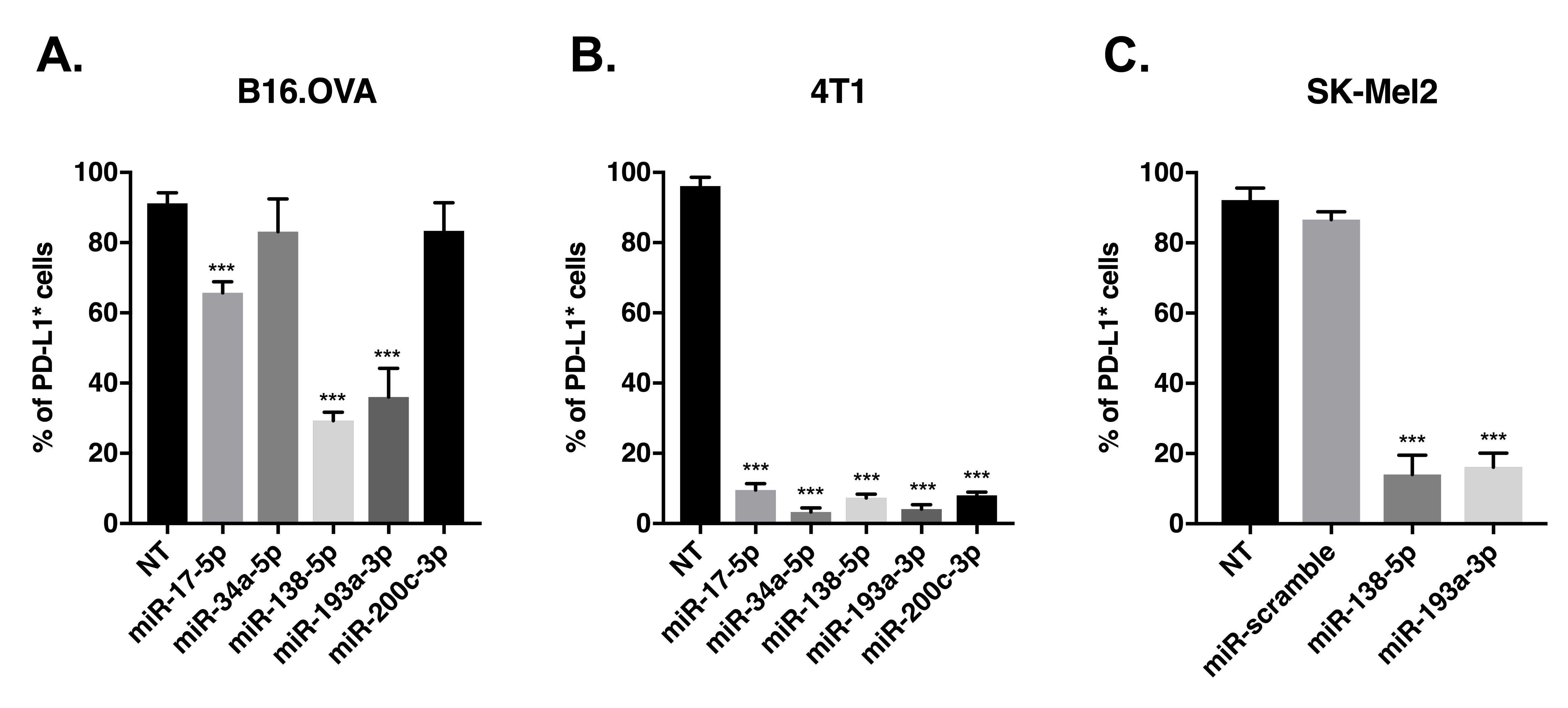

Supplement: S2 Fig — Effect of selected miRNA mimics on PD-L1 expression in 4T1 (A), B16.OVA (B) and SK-Mel2 (C). B16.OVA cells and 4T1 cells were transfected with miR-17-5p, miR-34a-5p, miR-138-5p, miR-193a-3p or miR-200c-3p and 48 hours post-infection, cells were harvested and PD-L1 protein levels were analysed by flow cytometry using antibody against murine PD-L1. SK-MEL2 cells were transfected with miR-138-5p, miR-193a-3p or miR-scramble as a control and 48 hours post-infection, cells were harvested and PD-L1 protein levels were analysed by flow cytometry using antibody against human PD-L1. Data are presented as mean ± SEM of three independent experiments performed in triplicate. One-way ANOVA followed by Dunnett’s multiple comparisons test. ** p-value < 0.01 compared to control; *** p-value < 0.001 compared to control (NT, non-transfected cells). (TIFF) [file pone.0224072.s002.tiff]

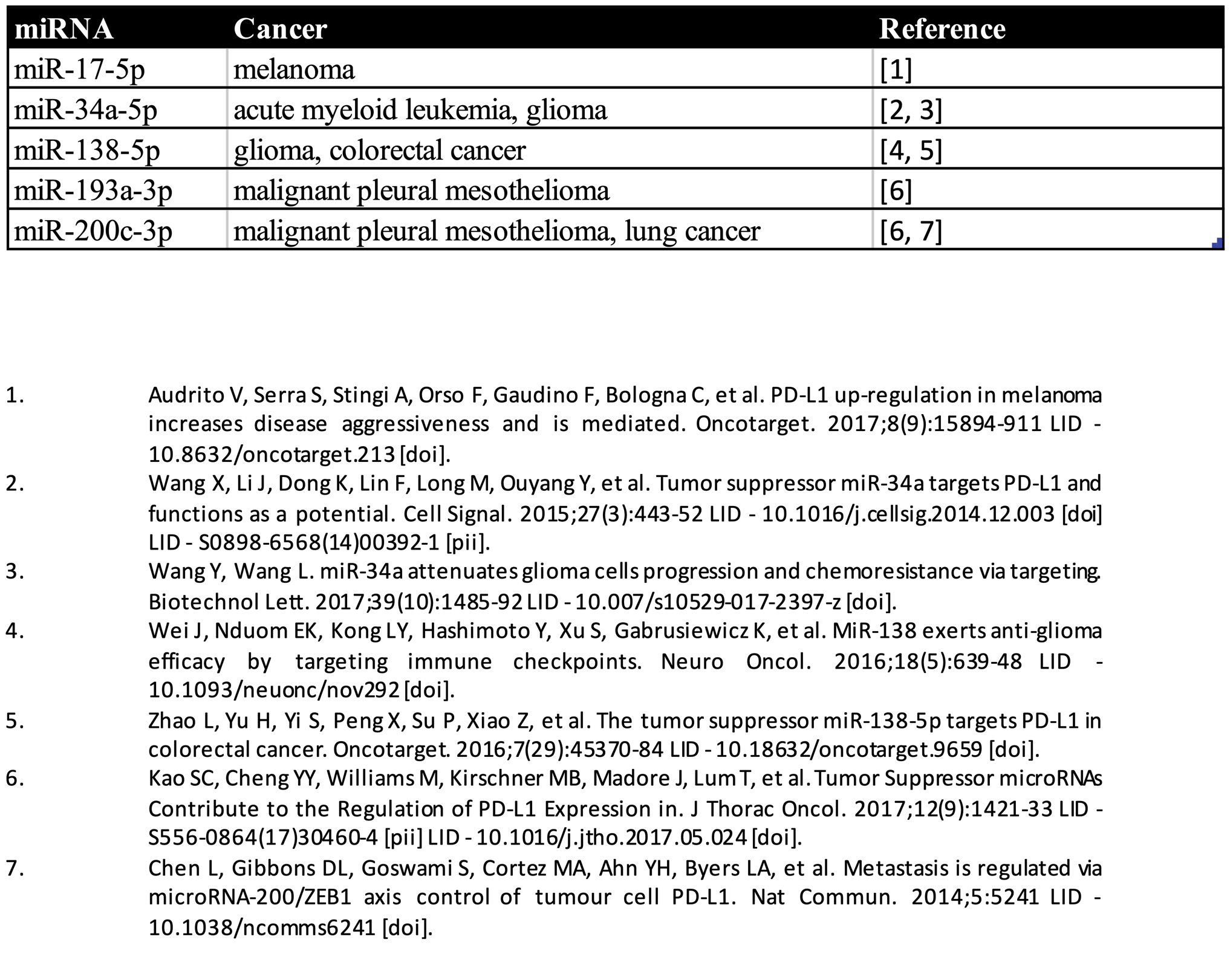

Supplement: S1 Table — (TIFF) [file pone.0224072.s003.tiff]
